# Computed tomography of the thorax in rabbits: a prospective study in ten clinically healthy New Zealand White rabbits

**DOI:** 10.1186/s13028-017-0340-x

**Published:** 2017-10-24

**Authors:** Désirée Müllhaupt, Sandra Wenger, Patrick Kircher, Nadja Pfammatter, Jean-Michel Hatt, Stefanie Ohlerth

**Affiliations:** 1 Clinic for Diagnostic Imaging, Vetsuisse Faculty, University of Zurich, Winterthurerstrasse 258c, 8057 Zurich, Switzerland; 2 Clinic for Zoo Animals, Exotic Pets and Wildlife, Vetsuisse Faculty, University of Zurich, Winterthurerstrasse 260, 8057 Zurich, Switzerland

**Keywords:** Computed tomography, Rabbit, Thorax, Thymus

## Abstract

**Background:**

Literature investigating the normal cross-sectional anatomy of rabbits with computed tomography (CT) is sparse and incomplete. The purpose of the present study was to investigate the normal thoracic structures, in particular the cranial thorax, with CT angiography in 10 clinically healthy New Zealand White (NZW) rabbits.

**Results:**

Absolute and relative measurements of the trachea, heart, thoracic caudal vena cava and aorta, right and left principal bronchi, right and left caudal lobar bronchi and the accompanying branches of the right and left pulmonary artery and vein, right and left lung volume and lung density were taken. The three lobes of the thymus (right ventral, right dorsal and left thoracic lobes) were identified in all rabbits. Both the right dorsal and left thoracic lobes of the thymus extended between the heart and thoracic wall in all individuals with the left lobe reaching more caudally in seven animals. Consequently, the craniocaudal extension of the left lung was smaller than the right lung in these rabbits. Volume of the left lung was significantly smaller than the right (P = 0.005). The cranial mediastinal, right and left tracheobronchial and the aortic thoracic lymph nodes were very small and identified in four, four, seven and ten rabbits, respectively. The heart took up a median of 4.0 intercostal spaces, and in seven rabbits, it was located in the 2nd–5th intercostal space. Median relative cardiac height and width measured 74 and 88%, respectively. The median angle of the trachea to the spine was 5°. Median density between the right and left lung did not significantly differ (− 549 and − 583 Hounsfield units, respectively). In all but one rabbit, atelectasis was present and classified as mild, moderate or severe in six, two and one individuals, respectively. Mild subclinical bronchopneumonia was diagnosed in seven rabbits.

**Conclusions:**

The present study provides species-specific anatomical CT information and reference values for structures in the thorax of the NZW rabbit. Subclinical bronchopneumonia appears to be a common CT finding.

## Background

The number of rabbits held as pets in European households has been increasing over the last years and rabbits have become frequent patients at veterinary clinics [1]. At the Clinic of Zoo Animals, Exotic Pets and Wildlife at the University of Zurich, rabbits are now the species presented most often [2]. In addition, rabbits are frequently used as laboratory animals and represent a common animal model to study respiratory diseases in humans.

In rabbits, respiratory diseases are a major cause of morbidity and mortality. Pasteurellosis, essentially caused by *Pasteurella multocida*, is the primary respiratory disease; however, many other bacterial and viral pathogens play a role in the disease complex [3]. In pet rabbits, infections with *Bordetella bronchiseptica* or *Staphylococcus* species are more common than an infection caused by *P. multocida* in rabbits from rabbitries [3]. Depending on the causative agent, clinical signs include upper respiratory disease (rhinitis, sinusitis, conjunctivitis, dacryocystitis), otitis, pleuropneumonia, bacteremia, pericarditis and abscesses in subcutaneous tissues, organs, bones, joints or genitalia [3]. Other reported thoracic diseases in the rabbit include bronchitis [4], thymoma [5], lymphoma [6], and metastatic pulmonary disease [7].

Because of its large availability, radiography represents the routine diagnostic imaging method in small animals to investigate abnormalities of the respiratory system, in particular the lower airway system. However, interpretation of thoracic radiographs in the rabbit presents several challenges. Among the intermediate-sized small companion mammals, the rabbit has a relatively small thoracic volume and the heart is located more cranially in the thorax. On latero-lateral radiographs, size of the retrosternal lucency, i.e. the cranial lung lobes is very small, making evaluation of the pulmonary opacity in the cranial thorax difficult. The accessory lobe of the right lung is also very small. Many rabbits have a large amount of subcutaneous and pericardial fat, further increasing overall thoracic opacity. Together, these species-specific features may simulate pulmonary disease or, on the contrary, mask true pulmonary changes [8, 9].

Computed tomography (CT) is considered the gold standard to evaluate thoracic disease in humans, and represents an increasingly available technology in veterinary medicine. Modern helical scanners allow very short scan times, thin slices, high resolution and post-processing software such as multiplanar reconstructions, making CT highly suitable for the examination of the thorax in small companion mammals. So far, CT has been reported to be superior to conventional radiographs in rabbits for the diagnosis of intrapulmonary disease such as abscesses or lung metastases [10]. So far, there is only a single report including an atlas comparing CT and cross-sectional cadaver anatomy of the rabbit’s neck and thorax. However, the study lacks measurements and information on the topical relationship of normal structures [11]. Another recent study evaluated in detail the normal CT anatomy and dimensions of the larynx and upper trachea in rabbits for the establishment of an animal model [12]. However, detailed cross-sectional studies of the normal lower airways in rabbits have not yet been performed. Therefore, the purpose of the present study was to use CT to investigate the normal thoracic structures, particularly the cranial thorax, in clinically healthy New Zealand White rabbits.

## Methods

### Animals

Ten clinically healthy 7-month-old intact female New Zealand White rabbits with a median body weight of 5.0 kg (SE 0.15 kg; range 4.4–6.0 kg) were included in the study. Animals were obtained from a wholesale source with annual hygiene monitoring and allowed to acclimatize to the new environment for 1 week. Rabbits were not fasted before CT.

### Anaesthesia

All rabbits were judged to be healthy based on a physical examination. Then, animals were sedated with a combination of fentanyl citrate 0.0473 mg/kg body weight (BW) and fluanisone 1.5 mg/kg BW (Hypnorm; VetaPharma Ltd, Leeds, UK) administered intramuscularly. Twenty minutes after the injection, they were placed and kept in sternal recumbency until the end of the CT scan. First, a venous catheter was placed in the external ear vein. Then, rabbits were pre-oxygenated for 2 min prior to induction of anaesthesia with propofol (Propofol 1% MCT; Fresenius Kabi AG, Oberdorf, Switzerland) given intravenously to effect, starting with a dose of 1 mg/kg BW over 20 s. Anaesthesia was maintained with a constant rate infusion of propofol (0.2–0.6 mg/kg BW/min). Oxygen was provided by a tight-fitting facemask. After the CT scan, animals received Ringer’s lactate solution 10 ml/kg BW (Ringer Lactat Fresenius; Fresenius Kabi AG, Oberdorf, Switzerland) intravenously over 30 min and were returned to the group when fully awake.

### Computed tomography

Examination of the thorax was performed in sternal recumbency using a 40-slice scanner (SOMATOM Sensation Open, Siemens Schweiz AG, Zurich, Switzerland). Images were acquired from the caudal cervical spine to the mid-abdomen (slice thickness 2 mm, increment 0.7 mm, pitch 1.2, 120 kVp, 120 mA, rotation time 1 s). To improve identification of cardiovascular structures, the thymus and lymph nodes, CT angiography was performed subsequently. Iodinated contrast agent (Ultravist^®^-370, Bayer AG, Zurich, Switzerland) was given intravenously as a manual bolus (dose 2 ml/kg BW). Bolus tracking was performed in the pulmonary outflow tract (threshold 100 Hounsfield units (HU)) with a subsequent caudocranial thoracic scan using a slice thickness of 2 mm. CT data was reconstructed to images with 0.75 mm slice thickness using a medium-frequency (soft tissue) and a high-frequency image reconstruction algorithm (lung, bone), respectively.

### Analysis of computed tomographic images

Image interpretation and all measurements were done with dedicated software using multiplanar reconstruction (MPR) images and electronic callipers integral to the software system (OsiriX Open Source™ 5.0.2, OsiriX Foundation, Geneva). Measurements were performed once and by the same author (DM). Bony structures, soft tissues and the lungs were evaluated in a bone (window width (WW) 1500 HU, window level (WL) 300 HU), soft tissue (WW 350 HU, WL 40 HU) and lung window (WW 1400 HU, WL– 500), respectively. Mediastinal, cardiovascular and bronchial structures were identified and measured on post-contrast images in an analogous manner to radiographs. If indicated, ratios were calculated to compensate for differences in size and body weight. Anatomic structures were identified based on standard literature [13–15]. The anatomical nomenclature used in this study followed the format of the Nomina Anatomica Veterinaria [16].

#### Measurements—mediastinal structures

In the sagittal plane, the maximal height of the caudal vena cava and the descending thoracic aorta were measured along their course. The length of the thoracic vertebra at the level of the tracheal bifurcation was determined along its mid-ventral surface to calculate ratios of the maximal height of the large vessels.

Location of the heart in relation to the intercostal spaces and number of intercostal spaces (ICS) determining cardiac width was determined subjectively based on MPR images. In the dorsal plane, the relative cardiac width was calculated as a ratio of the maximal cardiac width to the thoracic width at the same level. Relative cardiac height was calculated on sagittal images as the ratio of the maximal cardiac height measured along the ribs to the thoracic height at the same level. In the mid-sagittal plane of the caudal vena cava, the short axis of the heart was measured along the atrioventricular valves. Perpendicular to it, the long axis of the heart was measured from the ventral border of the tracheal bifurcation to the cardiac apex. The long and short axis dimensions were added, transposed onto the vertebral column and recorded as the number of vertebrae starting at the cranial edge of the 4th thoracic vertebra. The given distance along the vertebral column to the caudal point was estimated as the number of vertebrae resulting in a vertebral heart score [17, 18]. In the same plane, the cardiac angle was determined between the heart base and the apex, along the lumen of the left ventricle.

#### Measurements—airways, lungs

In the bone window, thoracic height was measured in the transverse plane at the level of and parallel to the 1st ribs between the ventral aspect of the 1st thoracic vertebra and the manubrium. At the same level, but in a lung window, the maximal luminal tracheal height and width were measured. A ratio of tracheal height to thoracic height at the thoracic inlet was calculated electronically. The cross-sectional area (CSA) of the tracheal lumen was measured just cranial to the tracheal bifurcation. Caudal to the tracheal bifurcation, the CSA of the lumen of the right and left principal bronchus were evaluated. At the level of the 6th rib, the CSA of the lumen of the caudal lobar bronchus and the accompanying branch of the pulmonary artery and vein were calculated on the right and left. In the dorsal plane, the interbronchial angle between the mid-axis of both principal bronchi was assessed. Lung density (HU) was determined on native images in a defined area of 50 mm^2^ dorsolateral to the right and left caudal lobar bronchi avoiding the inclusion of vessels. The tracheal angle was determined between the dorsal wall of the trachea and the spine in the sagittal plane.

The maximal cranial and caudal extent of the left and right lungs was determined. The volume of the right and left lungs was assessed delineating their cross-sectional areas on 8–11 transverse images at intervals of 1 cm from the cranial to the caudal thorax; volumes were calculated electronically.

#### Subjective evaluation of lung changes

Lung atelectasis was defined as loss of lung volume in combination with various degrees of increased attenuation in the ventral lung aspects. Atelectasis was classified subjectively in four grades for the right and left lung: no atelectasis; mild atelectasis: increased attenuation, ventral aspect of the lung partially aerated; moderate atelectasis: complete attenuation, less than to the level of the heart base; severe atelectasis: complete attenuation ≥ to the level of the heart base. Total grade of atelectasis referred to the most severely affected side.

Lung changes in the dorsal regions were classified according to a system described for the assessment of high-resolution CT findings in the dog [19]. Lung abnormalities were finally categorized subjectively in four grades: normal lung, mild (< 1/3 of the lung affected), moderate (1/3–2/3 affected), and severe (≥ 2/3 affected).

### Data analysis

Descriptive statistics and ratios were calculated electronically. Due to the small sample size, the nonparametric Wilcoxon signed rank test was used for the comparison of two related samples (SPSS statistics, release 23.0.0.2, IBM Corporation, Armonk, New York). A P value < 0.05 was considered significant.

## Results

Image quality was considered good to excellent in all rabbits despite minor motion artefacts due to spontaneous breathing. Branches of the lobar bronchi and the accompanying pulmonary vessels could always be identified up to the 3rd generation. Major abnormalities or pathologies were not detected in any animal. Therefore, all datasets were included in the study.

In eight and two rabbits, respectively, 12 and 13 thoracic vertebrae with an equivalent number of rib pairs were present; transitional vertebrae were not identified. The 1st to 9th rib pair was connected to the sternum whereas the 10th to 12th or 13th rib pair ended free and separately. The cartilages of the 7th to 9th rib were fused and joined uniformly the xyphoid process. The sternum was composed of the large manubrium cranially, the body including four vertebrae, and the xyphoid process caudally with a thin but broad cartilaginous process.

**Fig. 1:**
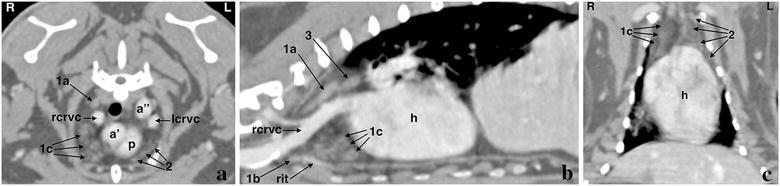
Post-contrast computed tomographic appearance of the thoracic thymus in a rabbit. **a** On a transverse image at the cranial border of the heart, the right dorsal thoracic thymus lobe (1a) is clearly isolated from the right ventral lobe (1c). The multilobulated heterogeneous structure of the caudal parts of the right ventral (1c) and left thoracic thymus lobes (2) is noted. A right (rcrvc) and left cranial vena cava (lcrvc) is present (ascending (a’) and descending thoracic aorta (a’’), pulmonary trunk (p)). **b** On a right sagittal image at the level of the right cranial vena cava (rcrvc), the separated right dorsal thoracic thymus lobe (1a) shows a round to ovoid shape with a compact structure. The right ventral thoracic thymus lobe has a small cranial (1b) and larger caudal (1c) part separated by the right internal thoracic artery (rit). Caudal to the right dorsal thoracic thymus lobe, the mediastinal lymph node (3) is seen (heart (h)). **c** On the dorsal plane, the left thoracic thymus lobe (2) extends more caudally in between the heart and the thoracic wall in comparison to the right ventral thoracic thymus lobe (1c). Cranial to the heart, only little lung tissue is seen on the right. Note the wide cardiac size in relation to the thoracic width (heart (h))

The thymus was visible pre- and postcontrast in the cranial and middle mediastinum in all rabbits (Fig. 1). All three lobes were identified: (1) the right dorsal thoracic lobe was seen dorsal to the right cranial vena cava; (2) the right ventral thoracic lobe was located ventral to the right cranial vena cava with two parts, cranial and caudal to the right internal thoracic artery, respectively; and (3) the left thoracic lobe was identified ventral to the left cranial vena cava with two parts, cranial and caudal to the left internal thoracic artery, respectively. Whereas the right dorsal thoracic lobe represented the most enhancing, homogeneous and compact lobe, which was easily identified in all rabbits, the right ventral and left thoracic lobes, in particular the caudal parts, subjectively demonstrated less enhancement, were less well marginated and more heterogeneous due to a loose structure and a mixed density composed of fat and soft tissue. The right dorsal lobe was of a round shape in the transverse plane and of an elliptical shape in the sagittal plane. It was clearly separated from the right ventral lobe. Both ventral lobes had a multilobulated appearance and extended caudally in between the heart and the thoracic wall. The cranial part of the left thoracic lobe was the smallest and most difficult lobe to visualize in all rabbits. However, the left lobe reached more caudally than the right lobe in seven animals (Fig. 1). In two rabbits, the caudal extent of the right and left thoracic lobe was at a similar level whereas the right thoracic lobe extended more caudally in one rabbit only. Multiple efforts were undertaken by the authors (DM, SO) to determine the total volume of the thoracic thymus and its lobes. However, due to its incoherent structure and very small caudal extensions in between the heart and the thoracic wall, reliable measurements could not be achieved.

**Fig. 2:**
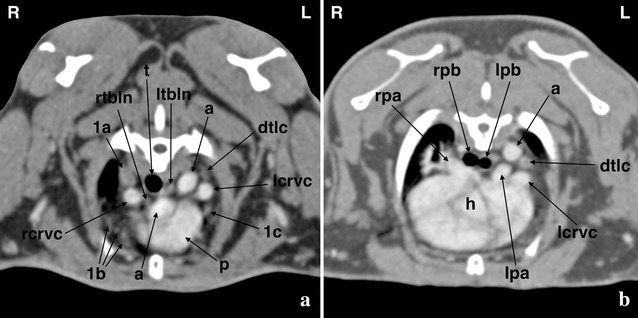
Post-contrast transverse computed tomographic images at the level of the pulmonary trunk (**a**) and the tracheal bifurcation (**b**) in a rabbit. The right (rtbln) and left tracheobronchial lymph nodes (ltbln) and one of the lymph nodes of the dorsal thoracic lymph centrum (dtlc) are shown (right dorsal thoracic thymus lobe (1a), right ventral thoracic thymus lobe (1b), left thoracic thymus lobe (1c), aorta (a), heart (h), trachea (t), pulmonary trunk (p), right (rpa) and left pulmonary artery (lpa), right (rpb) and left principal bronchus (lpb), right (rcrvc) and left (lcrvc) cranial vena cava)

The cranial mediastinal lymph node was identified caudomedial to the right dorsal thoracic lobe of the thymus in four individuals (Fig. 1b); it was very small in three of those four rabbits. The right and left tracheobronchial lymph nodes, located to either side of the caudal trachea just cranial to the bifurcation, were found in four and seven rabbits, respectively (Fig. 2); the left node was constantly larger than the right node and therefore easier to recognize. The dorsal thoracic lymph centrum i.e. the cranial and caudal aortic thoracic lymph nodes, was depicted in all rabbits lateral to the ascending and descending aorta comprising a single to multiple nodes of varying size (Fig. 2). In general, lymph nodes were round to slender in transverse and fusiform in sagittal images.

The oesophagus was identified on the left of the trachea at the thoracic inlet, then coursing dorsally to the trachea through the mediastinum, finally passing the diaphragm in the midline in all animals. It contained little to moderate amount of gas in six rabbits.

**Fig. 3:**
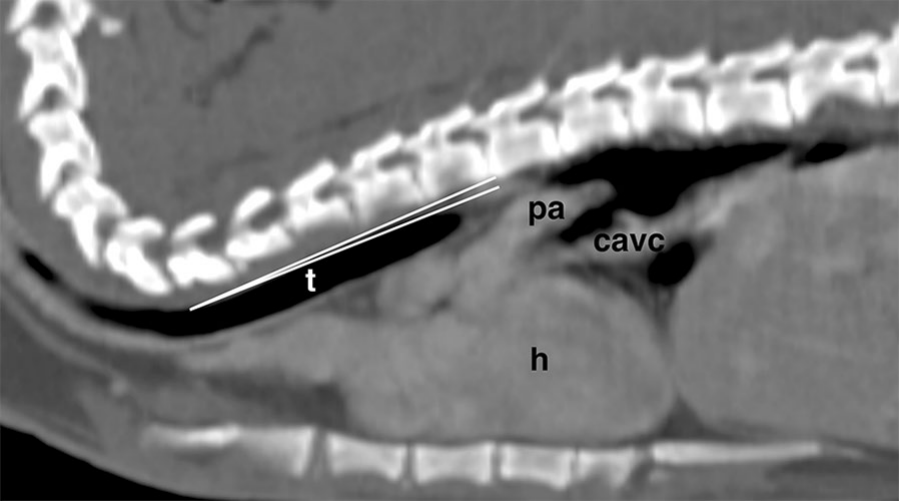
Post-contrast sagittal computed tomographic image in a rabbit (bone window): the tracheal angle between the dorsal wall of the trachea (t) and the thoracic spine is very small (white lines). Cardiac height in relation to the thoracic height is large (heart (h), caudal vena cava (cavc), pulmonary artery (pa))

The trachea was mildly mineralized and coursed almost parallel to the spine in all rabbits (Fig. 3). At the thoracic inlet, the tracheal cross section area was almost round. The tracheal bifurcation was located at the level of the 3rd or 4th thoracic vertebra in two and eight animals, respectively.

**Fig. 4:**
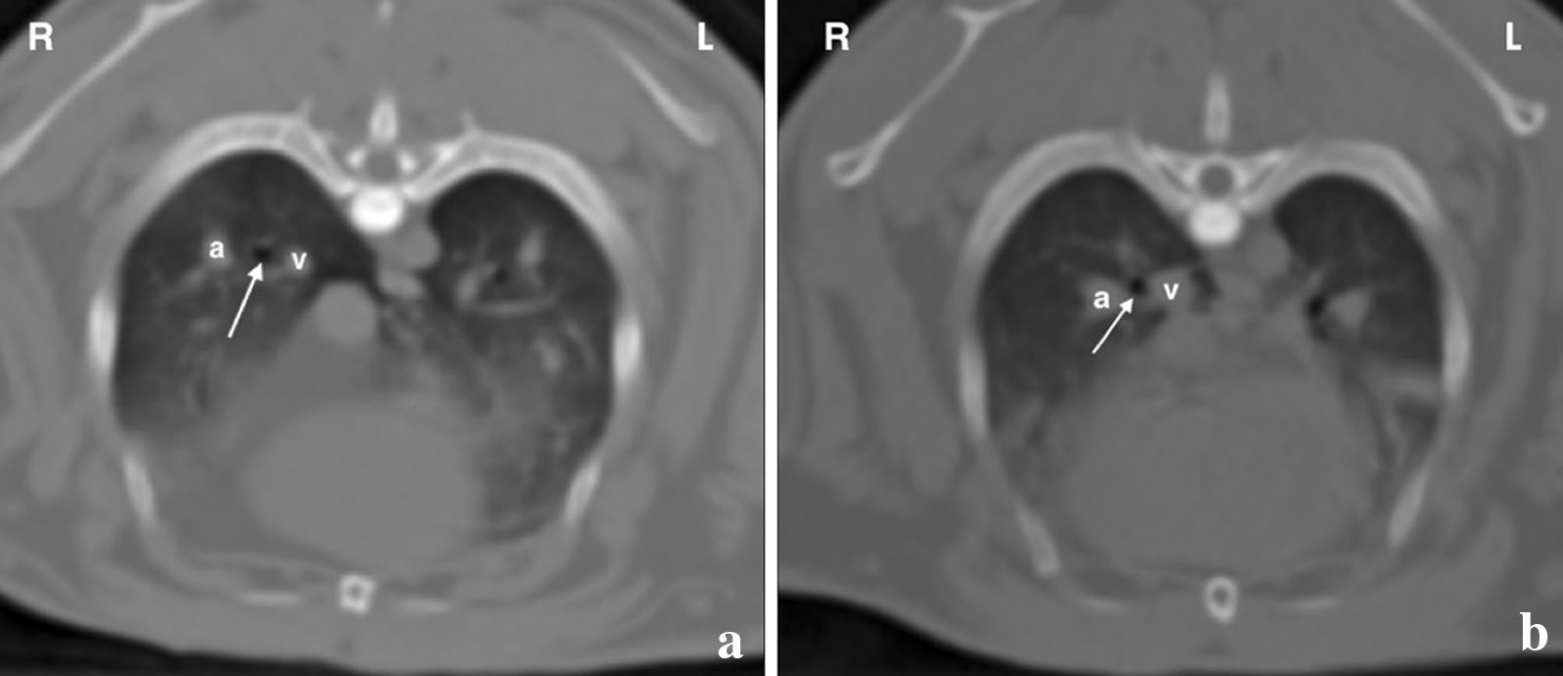
Post-contrast transverse computed tomographic images of the caudal lung lobes in a rabbit. **a** The branches of the pulmonary artery (a) and vein (v) are seen lateral and medial, respectively, and in close relationship to the corresponding principal bronchi (arrow). **b** In contrast, the caudal lobar vessels are running parallel but with a certain distance to the lobar bronchi. The cross-sectional area of the bronchial tree is constantly smaller than the accompanying vessels throughout the lungs

The absolute and relative height of the aorta was significantly smaller than for the caudal vena cava (P = 0.03). Subjectively, the heart appeared globoid and large (Fig. 1), which was reflected by a high number of ICS and relative cardiac height and width. The heart took a median of 4.0 ICS (SD 0.33; mean 3.8; min 3; max 4) and was located more commonly in the 2nd–5th (n = 7) than in the 3rd–6th (n = 2) or 3rd–5th (n = 1) ICS. In the sagittal plane, the cardiac axis was mildly oriented from craniodorsal to caudoventral in all rabbits. In the dorsal plane, the apex was located on the midline or to the left in six and four rabbits, respectively. In all rabbits, a left and right cranial vena cava was identified (Figs. 1, 2). Descriptive statistics of thoracic measurements are provided in Table 1. The CSA of the caudal lobar branch of the pulmonary artery was of a similar size as the accompanying vein bilaterally (P = 0.2 and 0.8); on the other hand, CSA of both, the lobar venous and arterial branch, was seven times the median CSA of the adjacent lobar bronchi (Fig. 4). Subjectively, CSA of the bronchial tree was constantly smaller than the accompanying vessels throughout the lungs of all rabbits.

**Table 1:** Descriptive statistics of thoracic computed tomographic measurements in 10 healthy New Zealand White rabbits

Variable	Median	Mean	SD	Min	Max
Cardiac angle (°)	35	35	2.5	30	38
Long axis of the heart (cm)	3.3	3.4	0.4	3	4
Short axis of the heart (cm)	3	3	0.2	2.7	3.2
Relative cardiac height (ratio maximal cardiac height/thoracic height) (%)	74	74	3.5	67	81
Relative cardiac width (ratio maximal cardiac width/thoracic width) (%)	88	88	4.9	81	95
Vertebral heart score	6.3	6.4	0.4	6	7.3
Maximal height of caudal vena cava (mm)	6.4	6.3	0.6	5	7.2
Maximal height of aorta (mm)	5.5	5.5	0.5	5	6.1
Ratio maximal height of caudal vena cava/length of thoracic vertebra at level of bifurcation (%)	73	71	5.9	57	76
Ratio maximal height of aorta/length of thoracic vertebra at level of bifurcation (%)	60	62	5.2	56	70
Angle of trachea to spine (°)	5	5	1.4	2	7
Tracheal height at thoracic inlet (mm)	5.4	5.1	1.1	2.4	6.2
Tracheal width at thoracic inlet (mm)	4.8	4.7	0.8	3.7	6.2
Ratio tracheal height/thoracic height at thoracic inlet (%)	26	25	5.5	10	30
CSA of tracheal lumen at bifurcation (mm^2^)	28.3	27.8	3.4	21.9	32.2
Luminal CSA of right principal bronchus (mm^2^)	15.5	15.4	2.4	11.1	18.8
Luminal CSA of left principal bronchus (mm^2^)	12.7	12.4	1.8	9.9	15.4
Luminal CSA of right caudal lobar bronchus (mm^2^)	1.7	2.0	0.6	1.3	2.9
Luminal CSA of left caudal lobar bronchus (mm^2^)	1.7	1.7	0.4	1.1	2.4
CSA of caudal lobar branch of right pulmonary artery (mm^2^)	14.8	14.4	4.9	7.7	22.3
CSA of caudal lobar branch of left pulmonary artery (mm^2^)	11.9	11.8	2.1	9	15.3
CSA of caudal lobar branch of right pulmonary vein (mm^2^)	15.8	15.1	4.7	7.7	22.5
CSA of caudal lobar branch of left pulmonary vein (mm^2^)	10.9	11.8	3.8	7.4	18.4
Interbronchial angle (°)	54	55	4.9	50	65
Lung volume right (cm^3^)	30.2	31.6	8.8	23.1	54
Lung volume left (cm^3^)	20.7	22.2	7.2	14.8	40.4
Density of right caudal lung (HU)	− 549	− 573	92	− 783	− 447
Density of left caudal lung (HU)	− 583	− 587	98	− 774	− 425

*SD* standard deviation; *Min* minimum; *Max* maximum; *CSA* cross-sectional area; *HU* Hounsfield unit

In agreement with the commonly shorter right thoracic lobe of the thymus, the right lung field was markedly longer than the left in the dorsal plane (Table 2). The right lung expanded cranially to the 3rd or 4th thoracic vertebra (T3 or T4) and never to T5. In contrast, the cranial border of the left lung was most often seen at T4 or T5. Caudally, the right lung reached most commonly T11 whereas the left lung expanded most frequently to T10. Also, in the transverse image just cranial to the heart, only the right cranial lung lobe was identified dorsally in four rabbits. In the other six animals, no lung tissue was seen at all at this level. The median right lung volume was significantly larger than the left (P = 0.005). The visceral pleura i.e. pleural fissure lines were rarely visible and location of the lung lobes was only possible based on the course of the lobar bronchi and the accompanying pulmonary vessels.

**Table 2:** Lung extent in 10 healthy New Zealand White rabbits on multiplanar computed tomographic images

Extent of right lung	N	Extent of left lung	N
T3–T9	1	T3–T11	1
T3–T10	2	T4–T9	3
T3–T11	2	T4–T10	3
T3–T12	1	T5–T10	2
T4–T11	4	T5–T11	1

*T* thoracic vertebra, *N* number of rabbits

In all rabbits, the branches of the pulmonary artery and vein were seen lateral and medial, respectively, and in close relationship to the corresponding principal bronchi (Fig. 4a). In contrast, the caudal lobar vessels were running parallel but with a certain distance to the lobar bronchi (Fig. 4b). Median luminal CSA of the right principal bronchus was significantly larger than on the left (P = 0.005). Although median CSAs of the caudal lobar branch of the right pulmonary artery and vein were larger than on the left, differences were not significant (P = 0.074 and 0.047, respectively). Otherwise, no significant differences were found between left and right measurements.

**Fig. 5:**
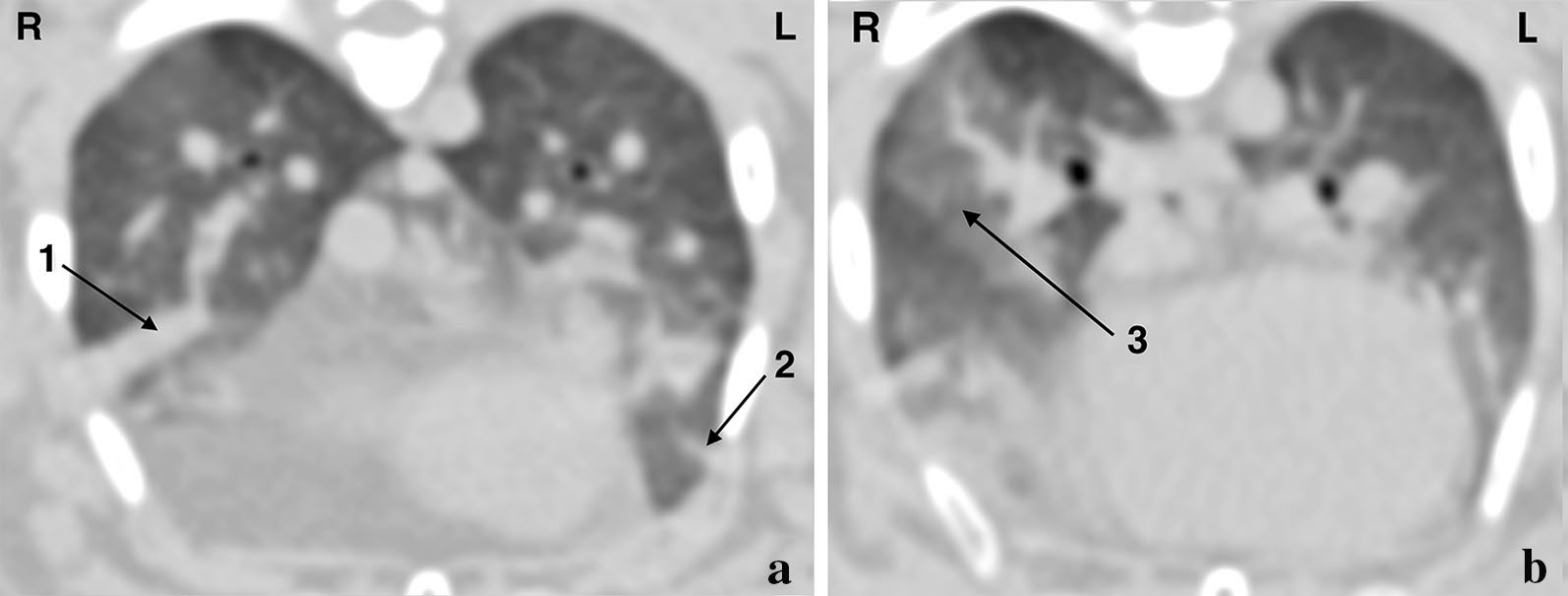
Post-contrast transverse computed tomographic images of a rabbit showing consolidation (1), a parenchymal band (2) (**a**) and ground glass opacity (3) (**b**) in the lung

In all but one animal, atelectasis was present and classified as mild, moderate and severe in six, two and one subjects, respectively. In three animals, the dorsal lungs were considered normal on CT. In the other seven rabbits, mild lung changes were diagnosed including ground glass opacity, parenchymal bands, consolidation, peribronchovascular thickening and/or pleural thickening (Fig. 5).

## Discussion

Although respiratory diseases are common in rabbits, clinical signs of lower respiratory diseases are often unspecific. The rabbit may present with anorexia, weight loss, depression and fatigue. Chronic infection in the thoracic cavity may be even subclinical long after the acute phase of the infection [3]. Low sensitivity of thoracic radiographs in rabbits further complicates the diagnosis for the treating veterinarian [3, 13]. Helical CT is considered highly superior to radiography for the evaluation of thoracic diseases, also in the small mammal patient. Rabbits also represent a common animal model to study respiratory diseases in humans. Due to a lack of pertinent data in the veterinary literature, the present study was performed to investigate the normal thoracic structures in clinically healthy New Zealand White rabbits using CT.

Radiographically, the craniocaudal dimension of the rabbit’s thorax has been described to be short with a small thoracic lung volume and a small cranial lung field, with a cranially located, relatively large heart [8, 9]. This was confirmed and more precisely defined with CT in the present study. Whereas on radiographs of the dog and cat [20] the cranial lung lobes extend to the thoracic inlet at end inspiration, the rabbits’ large cranial and middle mediastinum predominantly filled in the cranial thorax in the present study. However, the caudal extent of the lungs of the rabbits at the level of T9–T12 was similar or slightly less than that of the dog [21]. Relative cardiac height and in particular cardiac width and number of ICS were much higher in the rabbits than reported for dogs or cats [22]. This was also reflected in a small angle of the trachea to the spine in the present study. The more cranial location of the cardiac silhouette and the tracheal bifurcation in these rabbits is in contrast to a former radiographic report, where the carina lay more caudally, i.e. at the level of the 4th or 5th intercostal space [9]. Whereas the normal position of the cardiac apex in the dog and cat is always on the left in the ventrodorsal view/plane [22], it was found more often on the midline than to the left in the present study and therefore, appears more variable in the rabbit.

In the adult rabbit, the thymus persists and consists of a right ventral and dorsal thoracic lobe and a left thoracic lobe. According to an anatomic atlas of the rabbit [16], the left lobe expands more caudoventrally around the heart, whereas the right lobe stretches out further dorsally. Cranially, both lobes extend into the thoracic inlet. In the present study, all lobes were identified in the described locations in all animals. However, identification was challenging. The right dorsal thoracic lobe was identified most easily in all rabbits whereas the boundaries of the right ventral and left thoracic lobes were indistinct due to a multilobulated architecture interspersed with fat.

Values for tracheal height in relation to the thoracic height at the thoracic inlet were similar to those reported in non-brachycephalic dogs [23]. Similar to other species [20], the volume of the left lung was significantly smaller than for the right lung in these rabbits. This was already described in an anatomical study of the rabbit at the end of the 19th century [15]. Larger absolute sizes of the right lung lobes, the existence of the accessory lobe as well as a relatively larger left lobe of the thymus [14] represent possible explanations. Moreover, rabbits were not fasted for the present study, since this is not necessary in this species. Therefore, the full stomach on the left might have displaced the diaphragm cranially decreasing the volume of the left lung. Interestingly, the median CSA of the right principal bronchus was significantly larger than the CSA of the left principal bronchus; the median CSAs of the accompanying artery and vein were also larger on the right although borderline not significant. These findings could support an anatomical difference between the right and left lung. However, due to the small size of the structures, measurement errors should also be considered.

The mean lung density measured in the present study is markedly higher compared to the dog, where mean values of − 713 to − 846 HU were reported [24]. Animals were always in sternal and never in lateral recumbency and therefore, haemostasis and hypoventilation can be ruled out. Spontaneous superficial breathing, differences in body condition or compression of the lung secondary to a large abdomen may have caused this finding. However, subclinical pulmonary disease cannot be ruled out, and unfortunately, necropsy was not available in the present study. Atelectasis in the ventral lung aspects was a common finding in the present study and was most likely caused by sternal recumbency. Lung changes were classified according to a system described previously for the assessment of high resolution CT findings in the dog [19]. This classification system applied very well in the rabbits of this study where all kinds of linear and reticular opacities as well as ground glass opacity were observed. However, the presence of subclinical respiratory changes in these rabbits limits the significance of the results of the present study. This is reinforced by the relatively high lung density measurements in the present study where values were significantly higher than reported values for the normal human or canine lung [24, 25]. On the other hand, clinically unapparent lung changes are rather common in rabbits, and therefore, the results of the present study might just reflect the situation in a normal population.

The pulmonary vessels could be followed to the 3rd-degree branch in the rabbits of this study whereas they can be followed to the 4th-degree branch in the normal lung of dogs and cats [26]. Authors of this study hypothesize that this finding is most likely due to the smaller size of the animals and smaller diameter of their peripheral pulmonary vessels and presumably this small size creates problems due to the limits of image resolution. The CSA of the caudal lobar branches of the pulmonary arteries and veins were of a similar size, but 7 times the median CSA of the adjacent lobar bronchi. In dogs, the pulmonary artery CSA should be between 0.4 and 0.6 of the external CSA of the adjacent bronchus measured at the level of the T8 vertebra [27]. In the present study, it was not possible to obtain measurements at the level of T8, because branching of the lobar vessels and bronchi took place more cranially in all rabbits. However, since the CSA of the bronchial tree was smaller than the accompanying vessels throughout the lungs of all rabbits, we hypothesize that this finding may represent a species-specific feature.

As reported previously, an average number of 12 thoracic vertebrae and a left and right cranial vena cava appear to be common and species-specific in rabbits [14].

A few limitations have to be considered regarding this study. Although image quality was considered satisfactory, it could have been improved by scanning during apnea similar to the standard practice in dogs and cats. Attempts to assess accuracy or intra-, interobserver reproducibility of measurements were not undertaken. A post mortem examination including macroscopic and histopathological evaluation of the major organs was not performed and animals were considered to be healthy on a clinical examination only. Additionally, only a small number of animals of a single breed were included. Therefore, subsequent CT studies should be performed in a larger cohort of animals of various breeds, including pet rabbits. Comparison to post-mortem findings would be preferable.

## Conclusions

The present study provides preliminary species-specific computed tomographic anatomical information and reference values for structures in the thorax of the NZW rabbit. Subclinical bronchopneumonia appears a common CT finding.
